# Differential H_2_O_2_ Metabolism among Glioblastoma Subtypes Confers Variable Responses to Pharmacological Ascorbate Therapy Combined with Chemoradiation

**DOI:** 10.3390/ijms242417158

**Published:** 2023-12-05

**Authors:** Amira Zaher, Kranti A. Mapuskar, Jann N. Sarkaria, Douglas R. Spitz, Michael S. Petronek, Bryan G. Allen

**Affiliations:** 1 Department of Radiation Oncology, The University of Iowa, Iowa City, IA 52242, USA; amira-zaher@uiowa.edu (A.Z.); krantiashok-mapuskar@uiowa.edu (K.A.M.); douglas-spitz@uiowa.edu (D.R.S.); 2 Department of Radiation Oncology, Mayo Clinic, Rochester, MN 55905, USA; sarkaria.jann@mayo.edu

**Keywords:** glioblastoma, chemoradiation, glioblastoma subtypes, pharmacological ascorbate, antioxidant therapy, prooxidant, hydrogen peroxide, DNA damage

## Abstract

Glioblastoma (GBM), a highly lethal and aggressive central nervous system malignancy, presents a critical need for targeted therapeutic approaches to improve patient outcomes in conjunction with standard-of-care (SOC) treatment. Molecular subtyping based on genetic profiles and metabolic characteristics has advanced our understanding of GBM to better predict its evolution, mechanisms, and treatment regimens. Pharmacological ascorbate (P-AscH^−^) has emerged as a promising supplementary cancer therapy, leveraging its pro-oxidant properties to selectively kill malignant cells when combined with SOC. Given the clinical challenges posed by the heterogeneity and resistance of various GBM subtypes to conventional SOC, our study assessed the response of classical, mesenchymal, and proneural GBM to P-AscH^−^. P-AscH^−^ (20 pmol/cell) combined with SOC (5 µM temozolomide and 4 Gy of radiation) enhanced clonogenic cell killing in classical and mesenchymal GBM subtypes, with limited effects in the proneural subtype. Similarly, following exposure to P-AscH^−^ (20 pmol/cell), single-strand DNA damage significantly increased in classical and mesenchymal but not proneural GBM. Moreover, proneural GBM exhibited increased hydrogen peroxide removal rates, along with increased catalase and glutathione peroxidase activities compared to mesenchymal and classical GBM, demonstrating an altered H_2_O_2_ metabolism that potentially drives differential P-AscH^−^ toxicity. Taken together, these data suggest that P-AscH^−^ may hold promise as an approach to improve SOC responsiveness in mesenchymal GBMs that are known for their resistance to SOC.

## 1. Introduction

Glioblastoma (GBM) is the most common and aggressive adult primary brain malignancy, with approximately 14,000 cases diagnosed annually [1,2,3,4]. GBM has poor clinical outcomes, with a 5-year overall survival rate of less than 10% [1,2,3]. The current standard of care (SOC) for GBM consists of maximum safe surgical resection followed by radiation therapy and temozolomide (TMZ) [3]. Unfortunately, effective treatment options for GBM are faced with multiple challenges, including radiation resistance and radiation-induced brain injury, the blood–brain barrier, and tumor heterogeneity. GBM tumors are often resistant to radiation due to altered metabolism, hypoxia, and increased DNA repair capacity [5,6]. Moreover, radiation therapy is known to induce a variety of injuries to the brain, ranging from neuroinflammation and cognitive dysfunction to dementia [7,8]. The blood–brain barrier is altered in GBM due to the interference of tumor vasculature; this alteration contributes to hypoxia and confers resistance to chemotherapeutic agents such as TMZ [9,10,11]. These challenges underscore the urgent need for innovative approaches to sensitize GBM to SOC therapy.

Recently, pharmacological ascorbate (high-dose intravenous infusions of vitamin C resulting in plasma concentrations of ≈20 mM, P-AscH^−^) has garnered attention as a potential anticancer agent to enhance the response to SOC therapy across various malignancies, including GBM [12,13,14,15,16]. When ascorbate is oxidized, it generates high levels of hydrogen peroxide (H_2_O_2_), which can react with redox-active iron, primarily Fe^2+^, through Fenton chemistry, resulting in an excess of damaging hydroxyl radicals capable of disrupting biological macromolecules [12,13,16,17]. Since malignant cells are believed to exhibit altered mitochondrial function, leading to increased steady-state levels of reactive oxygen species, increased iron concentrations, and reduced levels of antioxidant enzymes (e.g., catalase), P-AscH^−^ is hypothesized to be a selective pro-oxidant in cancer cells relative to their non-malignant counterparts [16,17,18]. Recently, a phase 2 clinical trial demonstrated that P-AscH^−^ enhanced SOC clinical outcomes in GBM patients increasing overall survival to 19.6 months [16,19]. However, a subset of subjects receiving P-AscH^−^ was unresponsive, prompting the need to identify biologically relevant factors driving P-AscH^−^ response.

GBM subtypes, classified based on their transcriptional profiles into mesenchymal, classical, and proneural subtypes, offer a potential avenue for refining GBM therapy. While IDH mutational status and O^6^-methylguanine-DNA methyltransferase (MGMT) methylation are primary considerations in GBM treatment, GBM molecular subtypes are currently underemphasized in patient evaluation. Nonetheless, earlier studies revealed that GBM subtypes exhibit distinct clinical outcomes, metabolic characteristics, and immune signatures [20,21]. Additionally, it has been observed that patient prognosis varies among subtypes, with proneural and classical GBMs demonstrating a more favorable outlook compared to mesenchymal tumors [20,21,22]. Consequently, these subtype-related distinctions present an opportunity to guide the utilization of P-AscH^−^ therapy in GBM. This study aimed to conduct the first preclinical assessments of the response of mesenchymal, classical, and proneural GBM subtypes to P-AscH^−^ therapy, while also investigating potential disparities in H_2_O_2_ metabolism contributing to their varying responses, thus providing a novel insight into GBM H_2_O_2_ metabolism in the context of P-AscH^−^ therapy.

## 2. Results

### 2.1. Glioblastoma Subtypes Exhibit a Differential Response to P-AscH^−^ with SOC

**Figure 1:**
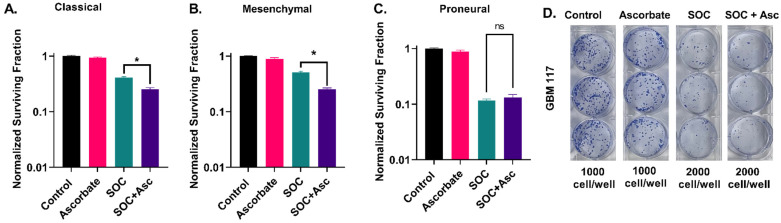
The effect of P-AscH^−^ on colony formation in different GBM subtypes treated with SOC. (**A**). P-AscH^−^ significantly enhanced SOC in classical GBM. Data are shown from GBM06 and GBM76. (**B**). SOC in mesenchymal GBM was significantly enhanced by the addition of P-AscH^−^. Data presenting U87, U251, and GBM39. (**C**). P-AscH^−^ did not show differences in response to SOC in proneural GBM. Data are shown from GBM117 and GBM85. (**D**). Representative images showing GBM117 colonies treated with SOC and SOC + P-AscH^−^. * is statistically significant, *p* < 0.05. ns, not significant. Data show normalized survival fractions from 3 replicates in each cell line. Error bars represent the standard error of the mean (SEM).

The cytotoxic effect of combining P-AscH^−^ with SOC (5 µM TMZ and 4 Gy IR) in the three GBM subtypes was initially assessed using a colony formation assay (Figure 1D). The addition of P-AscH^−^ significantly augmented the cytotoxicity of SOC in classical GBM cells compared to SOC treatment alone (mean = 0.25, 0.41, respectively, *p* = 0.001; see Figure 1A and Figure S1A,B). Interestingly, mesenchymal GBMs, often associated with treatment resistance, displayed a substantial enhancement in cell killing when treated with SOC + P-AscH^−^ compared to SOC alone (means = 0.25, 0.51, respectively, *p* = 0.0009, Figure 1B and Figure S1C–E). The change in cell survival between SOC and SOC + P-AscH^−^ was more pronounced in mesenchymal GBM cells than in classical GBM cells (ΔNSF = 0.26, 0.16, respectively). These findings suggest that mesenchymal GBMs are more sensitive to P-AscH^−^ therapy compared to classical GBMs. P-AscH^−^ did not enhance the cytotoxic effects of SOC in proneural GBMs (Figure 1C,D and Figure S1F,G). These data underscore the distinct responses to P-AscH^−^ observed within different GBM subtypes.

### 2.2. DNA Damage Responses following P-AscH^−^ Treatments Are Subtype-Dependent

DNA damage is a recognized primary mechanism for cell destruction induced by both radiation and temozolomide. Therefore, the impact of P-AscH^−^ on the induction of DNA damage was evaluated within this model system. Previous research showed that P-AscH^−^ induces single-strand DNA breaks through site-specific oxidations via hydroxyl radicals [23,24,25]. Thus, it was hypothesized that the induction of single-stranded DNA damage by P-AscH^−^ might occur in a subtype-specific pattern, similar to the in vitro enhancement of SOC.

**Figure 2:**
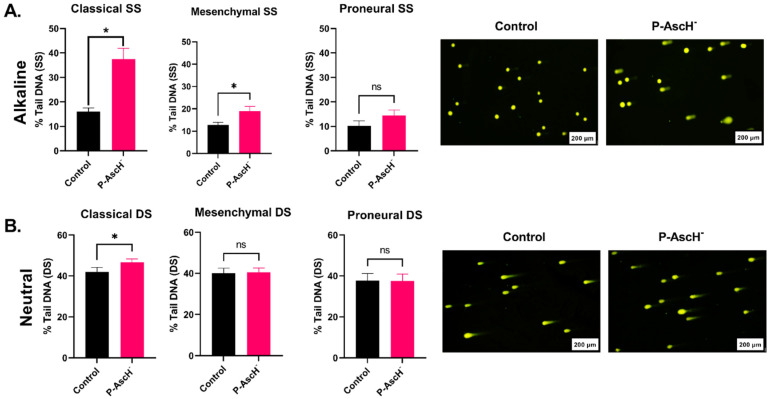
DNA damage following 1 h treatment with 20 pmol/cell P-AscH^−^. Classical (GBM06 and GBM76), mesenchymal (U87, U251, GBM39), and proneural (GBM117) data are shown. (**A**). Single-strand DNA breaks as % tail DNA in GBM subtypes following P-AscH^−^ treatment using the alkaline comet assay. Representative images (right) showing alkaline comets in GBM06 cells. (**B**). Double-strand breaks as % tail DNA in GBM subtypes following P-AscH^−^ treatment using the neutral comet assay. Representative images (right) showing neutral comets in GBM117 cells. The scale on all representative images is 200 µm. * is statistically significant, *p* < 0.05. ns, not significant. Data are shown from 3 replicates in each cell line. Error bars represent the standard error of the mean (SEM).

Consistent with the observed cell-killing effects (Figure 1), P-AscH^−^ significantly increased single-stranded DNA damage in both classical and mesenchymal GBM cells. This effect was not observed in proneural GBMs (*p* = 0.006, 0.017, and 0.24, respectively; Figure 2A and Figure S2A–E). Notably, P-AscH^−^ induced the highest level of single-strand DNA breaks in classical GBM when compared to mesenchymal and proneural GBMs (37.5%, 18.9%, and 14.4%, respectively; Figure 2A and Figure S2A–F). These results align with the subtype-specific enhanced cell-killing effects of P-AscH^−^ and emphasize the diverse responses within different GBM subtypes. We also assessed the induction of double-stranded DNA breaks. Classical GBMs showed a significant increase in double-stranded DNA breaks (*p* = 0.018) with P-AscH^−^ (Figure 2B and Figure S3C). In contrast, this effect was not observed in mesenchymal or proneural GBMs (*p* = 0.78, and 0.97, respectively; Figure 2B and Figure S3A,B,D,F). These results align with the subtype-specific enhanced cell-killing effects of P-AscH^−^ and underscore the potential significance of DNA damage as a central mechanism of action.

### 2.3. GBM Subtypes Exhibit Subtype-Dependent Differences in Hydrogen Peroxide Metabolism

H_2_O_2_ is considered the primary mediator of P-AscH^−^ cancer cell toxicity due to its ability to react with redox-active iron, leading to DNA oxidative damage [16,17,26,27,28,29]. Since P-AscH^−^ has been demonstrated to enhance the cell-killing effects of SOC and induce DNA damage in a subtype-dependent manner (Figure 1 and Figure 2), we hypothesized that H_2_O_2_ metabolism varied amongst the three GBM subtypes.

**Figure 3:**
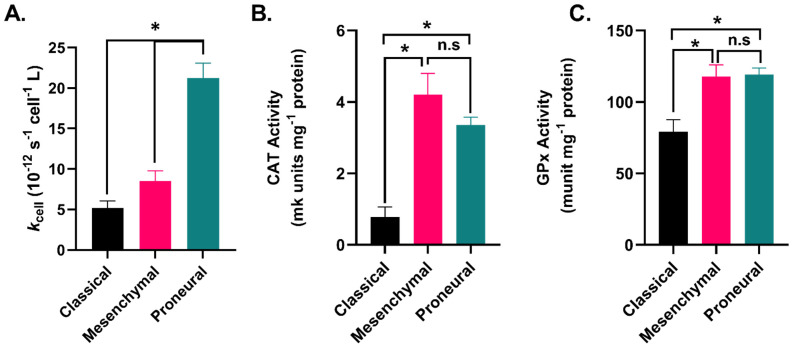
Differential H_2_O_2_ metabolism in GBM subtypes. (**A**). Hydrogen peroxide removal rates (10^−12^ s^−1^ cell^−1^ L) in GBM subtypes). Classical (GBM06 and GBM76), mesenchymal (U87, U251, GBM39), and proneural (GBM117 and GBM117) data are shown (**B**). Catalase activity (mk units per mg protein) in GBM subtypes. Classical (GBM06 and GBM76), mesenchymal (U251 and GBM39), and proneural (GBM117) data are shown. (**C**). GPx1 activity (munit per mg protein) in GBM subtypes. Classical (GBM06 and GBM76), mesenchymal (U251 and GBM39), and proneural (GBM117) data are shown. Data are shown from 3 replicates per cell line. * is statistically significant, *p* < 0.05. ns, not significant. Data are shown from 3 replicates in each cell line. Error bars represent the standard error of the mean (SEM).

The classical subtype had the lowest rate of H_2_O_2_ removal (5.2 × 10^−12^ s^−1^ cell^−1^ L) in comparison to both mesenchymal (8.5 × 10^−12^ s^−1^ cell^−1^ L, *p* = 0.476), and proneural cells (21.2 × 10^−12^ s^−1^ cell^−1^ L, *p* = 0.0001; Figure 3A and Table S1). Given the observed differences in the rate of H_2_O_2_ removal amongst the subtypes, we examined the enzymatic activity of both catalase (Cat) and glutathione peroxidase (GPx). Cat and GPx both play central roles in H_2_O_2_ metabolism. No significant differences were observed in the mesenchymal and proneural cells in either Cat (*p* = 0.52) or GPx (*p* = 0.99) activity (Figure 3B,C). However, classical GBMs showed significantly lower CAT activity in comparison to both mesenchymal and proneural GBM cells (*p* = 0.0003, and 0.0124, respectively; Figure 3B,C and Figure S4A,B). Significantly lower GPx activity was also observed in classical GBM cells compared to both mesenchymal and proneural cells (*p* = 0.01 and 0.03, respectively; Figure 3B,C and Figure S4A,B). Following the assessment of H_2_O_2_ metabolism in GBM subtypes, it appears that they have different capacities to remove H_2_O_2_ which can be attributed to the differential activities of Cat and GPx among the different subtypes. However, it is noteworthy that the differences in H_2_O_2_ metabolism between mesenchymal and proneural GBM may not be the sole factor responsible for mediating P-AscH^−^ toxicity and inducing DNA damage.

### 2.4. Increased P-AscH^−^ Dosing Further Improves Overall Survival in a Murine Xenograft Model of Mesenchymal GBM

Mesenchymal GBMs are well known for their aggressive nature and resistance to SOC treatment [30,31]. Since P-AscH^−^ significantly improves the response to SOC and induced DNA damage in mesenchymal GBM cells in vitro, we postulated that P-AscH^−^ could enhance SOC therapy in vivo and that the frequency of P-AscH^−^ dosing played a crucial role in response. We conducted a study comparing the overall survival of mice bearing mesenchymal U87 xenografts treated with SOC ± P-AscH^−^ using two separate P-AscH^−^ dosing schedules as outlined in Table 1. Sequence 1 dosed P-AscH^−^ three days per week, while Sequence 2 dosed ascorbate five days per week.

**Figure 4:**
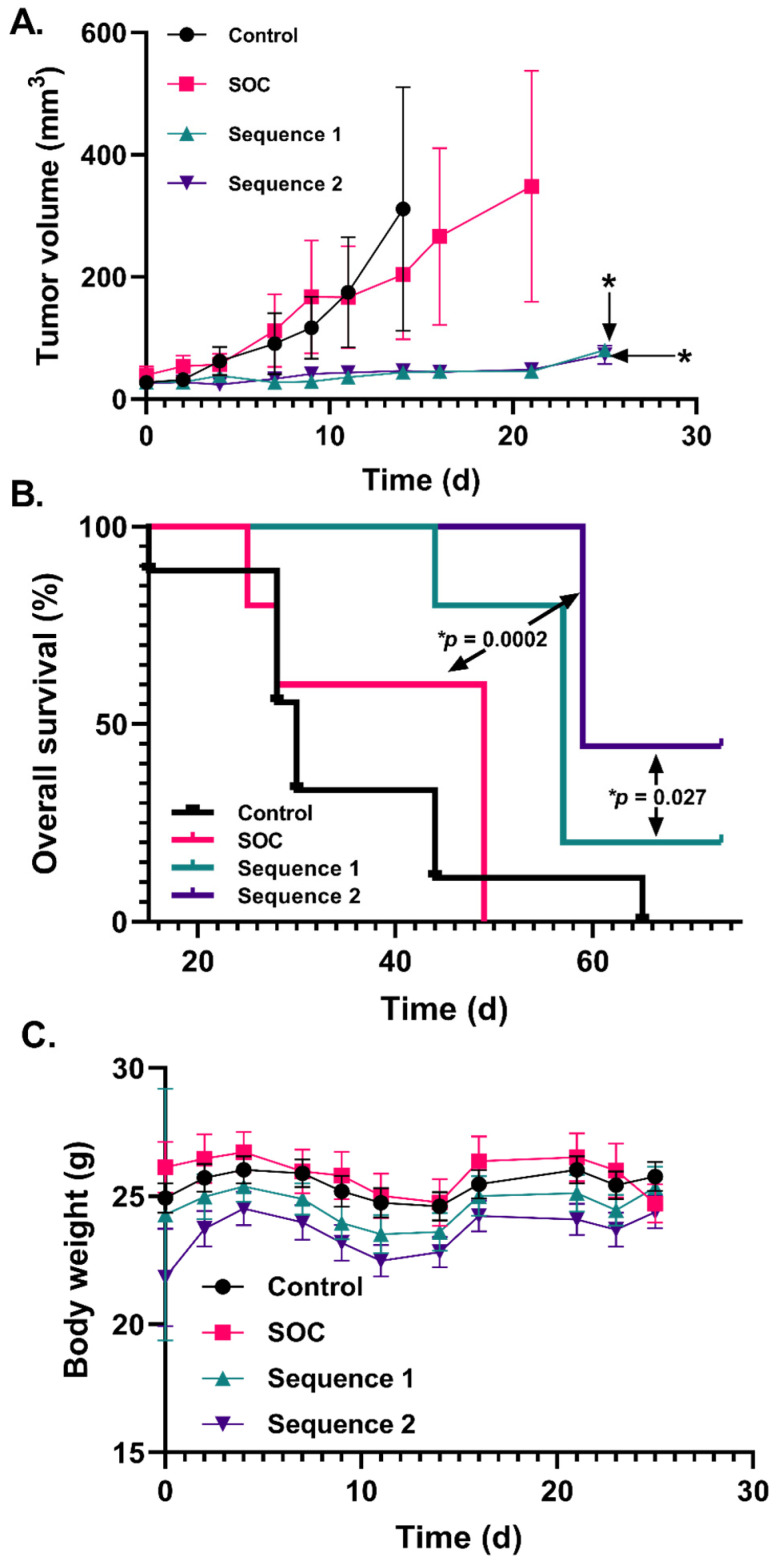
Increasing P-AscH^−^ doses in mesenchymal GBM further enhances overall survival in a xenograft mouse model. Control (n = 10), SOC (n = 6), Sequence 1 (n = 5), Sequence 2 (n = 9). (**A**). Tumor growth rate of U87 xenografts with SOC compared to P-AscH^−^ 3 times/week and 5 times/week. (**B**). Percent overall survival in mice with U87 tumors following treatment with the addition of P-AscH^−^ 3 days per week or 5 days per week. (**C**). Body weights in grams (g) of mice in the control group and the 3 treatment groups showed no significant changes in body weight. * is statistically significant, *p* < 0.05. Error bars represent the standard error of the mean (SEM).

Both Sequence 1 and Sequence 2 resulted in a significant reduction in tumor growth rates compared to SOC treatment alone (*p* = 0.005 and 0.0009, respectively). However, there were no significant differences in tumor growth between Sequence 1 and Sequence 2 (*p* > 0.9999; Figure 4A). Animals were observed for up to 66 days post treatment to assess overall survival. While there were no discernible differences in tumor growth between Sequence 1 and 2 (Figure 4A), Sequence 2 significantly improved overall survival when compared to both SOC alone and Sequence 1 (*p* = 0.0002 and 0.027, respectively; Figure 4B). Body weight changes across all groups followed a similar pattern (Figure 4C), with no notable body weight changes observed with additional P-AscH^−^ doses. These findings are in line with our previously reported data in an orthotopic U87 model, thus demonstrating the capacity of P-AscH^−^ to enhance SOC in both a flank and an orthotopic model [19]. Collectively, these data support the notion that P-AscH^−^ represents a promising strategy for enhancing SOC therapy and that increased dosing of P-AscH^−^ enhances GBM tumor control.

## 3. Discussion

P-AscH^−^ is hypothesized to exhibit antioxidant properties in non-malignant tissue while acting as a cytotoxic pro-oxidant agent in cancerous tissue [12,16,17,29]. Recent clinical data have shown promising outcomes for GBM patients treated with P-AscH^−^ [16,19]. Given the significance of these findings, this study aimed to gain insights into how different GBM subtypes respond to P-AscH^−^ therapy, potentially guiding future clinical applications of P-AscH^−^ in conjunction with SOC. We present evidence that P-AscH^−^ significantly enhances the response to SOC in classical and mesenchymal GBM, whereas this effect is not observed in proneural GBM. Our clonogenic survival data demonstrated that P-AscH^−^ enhances the response to SOC exclusively in classical and mesenchymal GBM cells, with both subtypes exhibiting significant increases in single-stranded DNA breaks. Only classical GBMs had a significant increase in double-stranded DNA breaks. Thus, classical GBM cells were the most sensitive to DNA damage induced by P-AscH^−^. Although P-AscH^−^ is primarily believed to affect DNA through the induction of single-strand breaks [23,24,25], it is conceivable that double-strand breaks are also created due to spontaneous generation or impaired DNA repair processes or close proximity of single-strand breaks, suggesting that classical GBMs may possess defective DNA repair machinery that promotes the persistence of single-strand breaks into double-strand breaks [32,33]. Interestingly, these findings reveal that P-AscH^−^ responses do not consistently align with the known prognostic outcomes of these subtypes, as proneural tumor cells often exhibit increased therapeutic sensitivity to SOC [21,22]. In summary, our study highlights the GBM subtype-specific effectiveness of P-AscH^−^ in vitro which is strongly associated with the induction of DNA damage.

To elucidate the differential responses to P-AscH^−^, we evaluated H_2_O_2_ metabolism in the three GBM subtypes. Proneural GBM cells had a high H_2_O_2_ removal rate along with elevated Cat and GPx activities. These results are consistent with proneural cell resistance to P-AscH^−^ therapy which can be attributed to their proficient H_2_O_2_ removal capacity. In contrast, classical GBM cells demonstrated decreased H_2_O_2_ removal rates and Cat and GPx activities compared to mesenchymal and proneural GBM cells, indicating an enhanced sensitivity to P-AscH^−^-induced DNA damage. This suggests that in classical GBM cells, Cat and GPx enzymes may play a more significant role in modulating responses to H_2_O_2_. Although mesenchymal GBM cells exhibited significantly lower H_2_O_2_ removal rates compared to proneural GBM, no differences were observed in their CAT and GPx activities. The distinct response patterns of these two subtypes may be attributed to other components of the antioxidant enzyme machinery, such as peroxiredoxins or superoxide dismutases [16,34]. Another contributing factor to the differential responses to P-AscH^−^ is the intracellular availability of Fe^2+^, which we have observed to be a major driver of P-AscH^−^ toxicity in GBM [16,35]. In aggregate, the differences in H_2_O_2_ metabolism between mesenchymal and proneural GBMs appear to be multifaceted, warranting further investigation to address other antioxidant enzymes and iron metabolic alterations. Mesenchymal GBMs are known for their poor prognosis due to their enhanced resistance to chemotherapy and radiotherapy as prior studies indicated that the transition from a proneural to a mesenchymal subtype promotes a therapy-resistant phenotype [30,31]. Our study demonstrated encouraging responses to P-AscH^−^ in mesenchymal GBM cells, which, when combined with SOC, increased susceptibility to P-AscH^−^-induced DNA damage.

Previously, GBM patients were administered 82.6 g ascorbate infusions three times a week, resulting in plasma ascorbate concentrations of ≈20 mM [16,19]. In this study, we utilized a previously established P-AscH^−^ dosing model (4 g kg^−1^) that was shown to enhance GBM response to standard of care therapy in flank and orthotopic GBM xenografts [16,19]. This study aimed to evaluate if P-AscH- dosing frequency had an impact on tumor control. The concentrations of AscH- achieved in the central nervous system through pharmacological dosing remain unclear; however, concentrations in the cerebrospinal fluid are known to be approximately 3.5–4.5 times greater than those observed in plasma [36,37,38]. Thus, it can be speculated that AscH^−^ concentrations in the cerebrospinal fluid following P-AscH- administration may reach ≥20 mM, which may be a key contributing factor to the successful application of P-AscH- in the context of GBM. In U87 tumors, we observed that increasing the frequency of P-AscH- administration with SOC from three days per week to five days per week during the active treatment phase significantly improved overall survival without inducing significant toxicities. These preclinical findings suggest that GBM patients with mesenchymal tumors may benefit from increased P-AscH^−^ dosing regimens, thus potentially guiding the design of future P-AscH^−^ clinical trials. Additionally, mesenchymal GBMs exhibit a unique and robust tumor microenvironment, enriched with tumor-associated macrophages [31,39], presenting another potential mechanism of P-AscH^−^ that has yet to be comprehensively explored in GBM [15]. In conclusion, this study highlights the potential role of the GBM subtype as a biomarker for P-AscH^−^ therapy. The observed variability in H_2_O_2_ metabolism across these subtypes necessitates further investigation, given the clinical demand for effective GBM treatment strategies.

## 4. Materials and Methods

### 4.1. Cell Lines

All GBM cells (Table 2) were cultured in DMEM-F12 media with the following additives: 15% FBS, 1% penicillin-strep, 1% Na-pyruvate, 1.5% HEPES, 0.1% insulin, and 0.02% fibroblast growth factor. Cells were maintained at 37 °C with 5% CO_2_. All experiments were performed at 4% O_2_ and 70–80% cell confluency.

### 4.2. Colony Formation Assay

To determine the effect of P-AscH^−^ in conjunction with SOC on reproductive integrity in the different GBM subtypes, classical, mesenchymal, and proneural cells were plated at 1–2 × 10^5^ cells per 60 mm dish. Within 24 h of plating, cells were serially treated with 5 µM temozolomide for 1 h and 20 pmol/cell P-AscH^−^ for 1 h and were irradiated with 4 Gy. Subsequently, cells from control or treated dishes were collected by treatment with trypsin (0.25%). Trypsin was inactivated with BR15 media containing 15% FBS and additives. Cell counts were made with a Coulter Counter and the cells were plated at various densities and allowed to grow for 1–4 weeks in complete media at 4% O_2_. Subsequently, the cells were stained with Coomassie Blue dye, colonies greater than 50 cells per plate were counted and recorded, and clonogenic cell survival was determined as described previously [40]. The surviving fraction was determined using the following equation:

$$Surviving\;Fraction=\frac{number\;of\;colonies\;counted}{number\;of\;cells\;plated}$$

Treatment groups were normalized to an untreated control to determine the normalized surviving fraction (NSF).

### 4.3. P-AscH^−^ Treatments

A 1M stock solution of ascorbic acid was used in all experiments. For in vitro studies, cells on a dedicated “count” dish were counted, and the number of cells per dish was used to calculate the volume of 1M ascorbic acid solution to treat the cells at 20 pmol/cell. For in vivo studies, the volume of ascorbic acid to be injected intraperitoneally was calculated based on a 4 g kg^−1^ mouse weight dose.

### 4.4. Neutral and Alkaline Comet Assays

Neutral and alkaline comet assays were carried out using the R&D CometAssay Electrophoresis Starter Kit (R&D, Minneapolis, MN) following the manufacturer’s instructions, with slight modifications. Briefly, cells were harvested immediately after P-AscH^−^ treatment and resuspended in DPBS at 2 × 10^5^ cells/mL. A total of 25 µL of cell suspension was mixed with agarose, and 50 µL of the mixture was spread on the well of a glass slide. Slides were dried in the dark at 4 °C for 10 min. Slides were then incubated in cold lysis buffer for 45 min at 4 °C. Following lysis, slides were incubated in a neutral buffer for 15 min or alkaline buffer for 20 min at room temperature. Slides were subjected to electrophoresis at 21 V in the appropriate buffer for 40 min for the neutral assay and 30 min for the alkaline assay. Slides were washed in water twice for 5 min, then in 70% ethanol for 5 min. Then, the slides were completely dried at 37 °C for 15 min. Slides were stained with 1X SYBR Gold (ThermoFisher, Waltham, MA, USA) for 30 min at room temperature in the dark. Excess stain was removed, and slides were briefly rinsed in water before allowing them to dry at 37 °C for 10 min. Fluorescent microscopy was used to image comets, and data were analyzed using the autoanalyzer software CometScore 2.0.0.38 to obtain percent tail DNA (http://rexhoover.com/index.php?id=cometscore, accessed: 21 December 2021).

### 4.5. Hydrogen Peroxide Removal Assay

The rate (k_cell_) of the removal of extracellular H_2_O_2_ by different subtypes of GBM was determined using the para-hydroxyphenylacetic acid (pHPA) plate reader assay as described previously [41,42]. A total of 50,000 cells from various subtypes of GBM were seeded per well of a 96-well (Costar Clear) bottom plate. Cells were incubated for at least 48 h at 37 °C, 4% O_2_, and 5% CO_2_. Subsequently, 10 uM hydrogen peroxide was added to wells and the system was quenched at predetermined times to determine the concentration of H_2_O_2_ remaining in the wells using a quenching solution of horseradish peroxide (HRP) that reacts with H_2_O_2_. This reaction of HRP with H_2_O_2_ activates HRP and subsequently oxidizes pHPA, resulting in a fluorescent dimer that corresponds to the H_2_O_2_ remaining in each well. Cells in each well were counted using a hemocytometer and the observed k_cell_ was determined.

### 4.6. Catalase Activity Assay

Catalase activity was determined using Abei’s method via UV-Vis spectroscopy, detecting the disappearance of H_2_O_2_ at 240 nm, ε240 = 39.4 M^−1^ cm^−1^ [43]. A total of 55.6 mM potassium phosphate buffer (pH 7.0) was used as the working buffer for this assay. The catalase reaction was initiated by adding an excess of 30 mM H_2_O_2_ to a final concentration of 10 mM in the assay cuvette. The absorbance of hydrogen peroxide was monitored immediately upon the start of the reaction for 120 s (s) at 10 s intervals. The natural log of the rate of H_2_O_2_ disappearance was then used to determine the kU of activity. Activity was then normalized to protein content in the sample as measured using the DC Protein Assay Kit (Bio-Rad, Hercules, CA, USA).

### 4.7. Glutathione Peroxidase Activity Assay

Glutathione peroxidase 1 (GPx) activity was determined spectrophotometrically using the method of Lawrence and Burk [44]. Briefly, in a buffer containing reduced glutathione (1 mM), glutathione reductase (1 E.U./mL), and NADPH (0.2 mM), activity in samples or standards was determined by measuring the disappearance of NADPH at 340 nm following the addition of H_2_O_2_ (final concentration = 0.25 mM). A unit of GPx activity was defined as 1 μmole of NAPDH oxidized/min. Activity was then normalized to protein content in the sample as measured using the DC Protein Assay Kit.

### 4.8. U87 Xenograft Murine Model

Female 6–8-week-old athymic nude mice (Envigo, Indianapolis, IN, USA) were used for this study. All procedures were approved by the University of Iowa Institutional Animal Care and Use Committee and conformed to NIH guidelines (IACUC protocol #0121207). Animals were kept at the University of Iowa animal care facility in a temperature-controlled environment with a 12 h light/12 h dark cycle. In total, 2 × 10^6^ U87 GBM cells were subcutaneously injected into the right flanks of nude mice. Upon the formation of palpable tumors, animals were randomly assigned to the following groups: control, SOC, Sequence 1, and Sequence 2 (Table 2). Ionizing radiation (IR) was delivered in 2 Gy fractions to the flank for a total of 12 Gy using the University of Iowa Xstrahl small animal radiation research platform. Temozolomide (TMZ) was delivered intraperitoneally at 2.5 mg kg^−1^. P-AscH^−^ was administered intraperitoneally at 4 g kg^−1^. Treatment consisted of two active treatment weeks followed by two adjuvant weeks. Dosing frequencies are shown in Table 1. A tumor volume > 1500 mm^3^ or tumors with necrotic ulceration were considered criteria for euthanasia.

### 4.9. Statistical Analysis

A one-way ANOVA with the Brown–Forsythe test was used to analyze colony formation assays, hydrogen peroxide removal assay, catalase activity, and GPx activity. Paired two-tailed t-tests were performed to analyze DNA damage data. Tumor growth rate and body weight data were analyzed using a two-way ANOVA. Overall survival data were analyzed on a Kaplan–Meier curve with the Gehan–Breslow–Wilcoxon test. GraphPad Prism 9 software was used for all statistical analyses.

## Figures and Tables

**Table 1:** Treatment groups, doses, and frequency of treatment.

Phase	Active Treatment (2 Weeks)			Adjuvant Phase (2 Weeks)		
Treatment	IR	TMZ	P-AscH^−^	IR	TMZ	P-AscH^−^
Control	-	-	-	-	-	-
SOC	2 Gy × 3 weekly.	2.5 mg kg^−1^ weekly	-	-	2.5 mg kg^−1^ weekly	-
Sequence 1	2 Gy × 3 weekly.	2.5 mg kg^−1^ weekly	4 g kg^−1^ × 3 weekly	-	2.5 mg kg^−1^ weekly	4 g kg^−1^ weekly
Sequence 2	2 Gy × 3 weekly.	2.5 mg kg^−1^ weekly	4 g kg^−1^ × 5 weekly	-	2.5 mg kg^−1^ weekly	4 g kg^−1^ weekly

**Table 2:** Characteristics of cell lines used in this study.

Cell Line	Source	Subtype	Sex	IDH Status	MGMT Methylation
U251	Millipore Sigma	Mesenchymal	M	Wild-type	Unmethylated
U87	ATCC	Mesenchymal	M	Wild-type	Unmethylated
* GBM06	Primary tumor	Classical	M	Wild-type	Unmethylated
* GBM76	Recurrent tumor	Classical	M	Wild-type	Methylated
* GBM39	Primary tumor	Mesenchymal	M	Wild-type	Methylated
* GBM117	Primary tumor	Proneural	M	Wild-type	Methylated
* GBM85	Primary tumor	Proneural	M	Wild-type	Methylated

* Cells obtained from the Mayo Clinic GBM patient-derived xenograft repository.

## Data Availability

Raw data are available upon request to the authors.

## Supplementary Materials

The following supporting information can be downloaded at: https://www.mdpi.com/article/10.3390/ijms242417158/s1.
