# Supplementary material for: Differential H2O2 Metabolism among Glioblastoma Subtypes Confers Variable Responses to Pharmacological Ascorbate Therapy Combined with Chemoradiation
Source: Int J Mol Sci. 2023 Dec 5;24(24):17158. doi: 10.3390/ijms242417158 (PMC10743151; doi:10.3390/ijms242417158)
Supplement: Supplementary file 1 [file ijms-24-17158-s001.zip › ijms-2725719-supplementary.pdf]

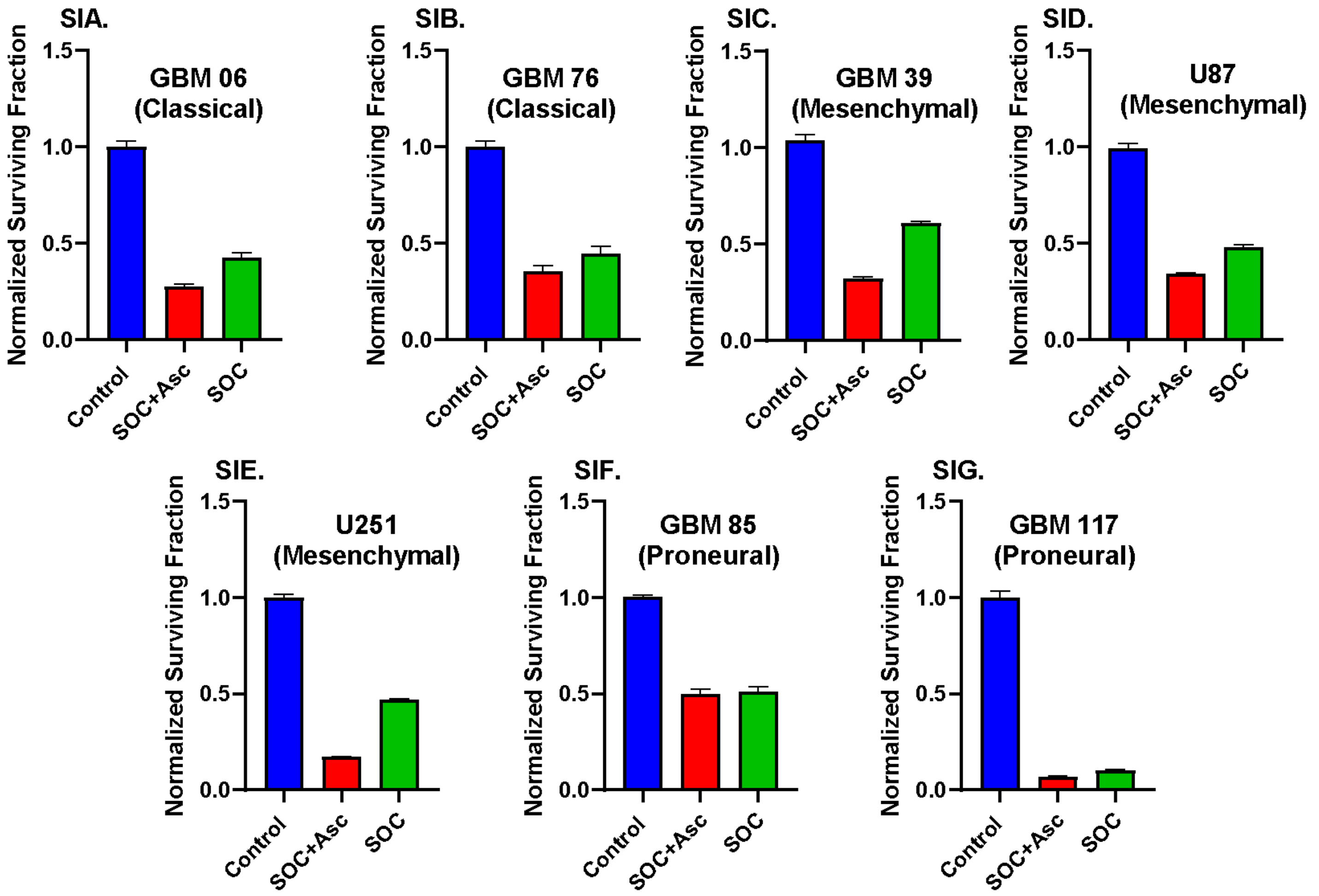

Figure S1. The effect of P-AscH$^-$ on response to standard of care (SOC) therapy in GBM cell lines. Data shown in classical, mesenchymal, and proneural GBM cells. Data are representing 3 replicates in each cell line (n = 3), GBM39 (n = 2). Error bars represent standard error of the mean (SEM).

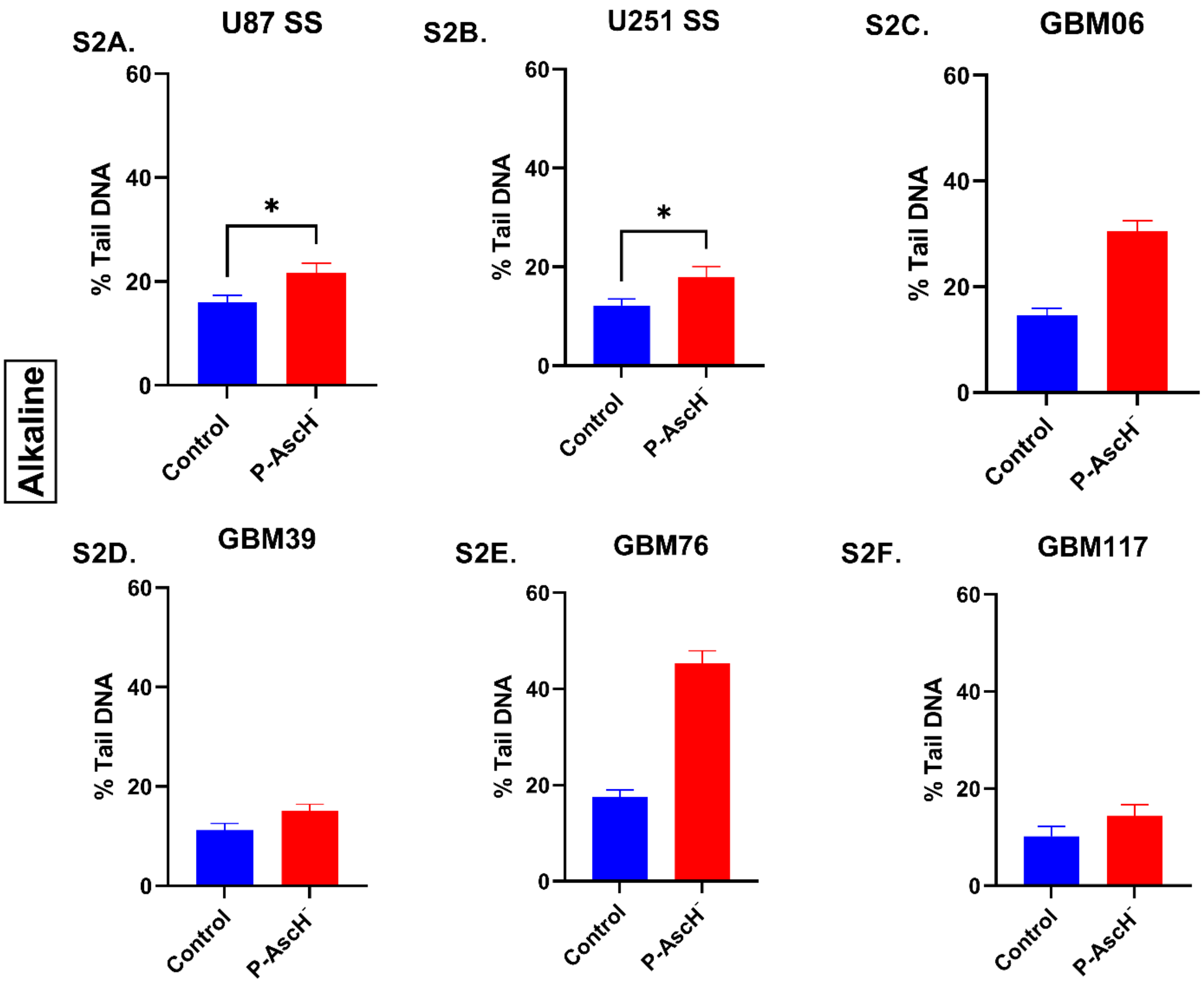

Figure S2. Single-strand DNA damage following P-AscH⁻ treatment in GBM cells. Single strand DNA damage as % Tail DNA following an alkaline comet assay in GBM cells treated with 20 pmol/cell P-AscH- for 1 hour. Data are representing 3 replicates in each cell line (n = 3). Error bars represent standard error of the mean (SEM).

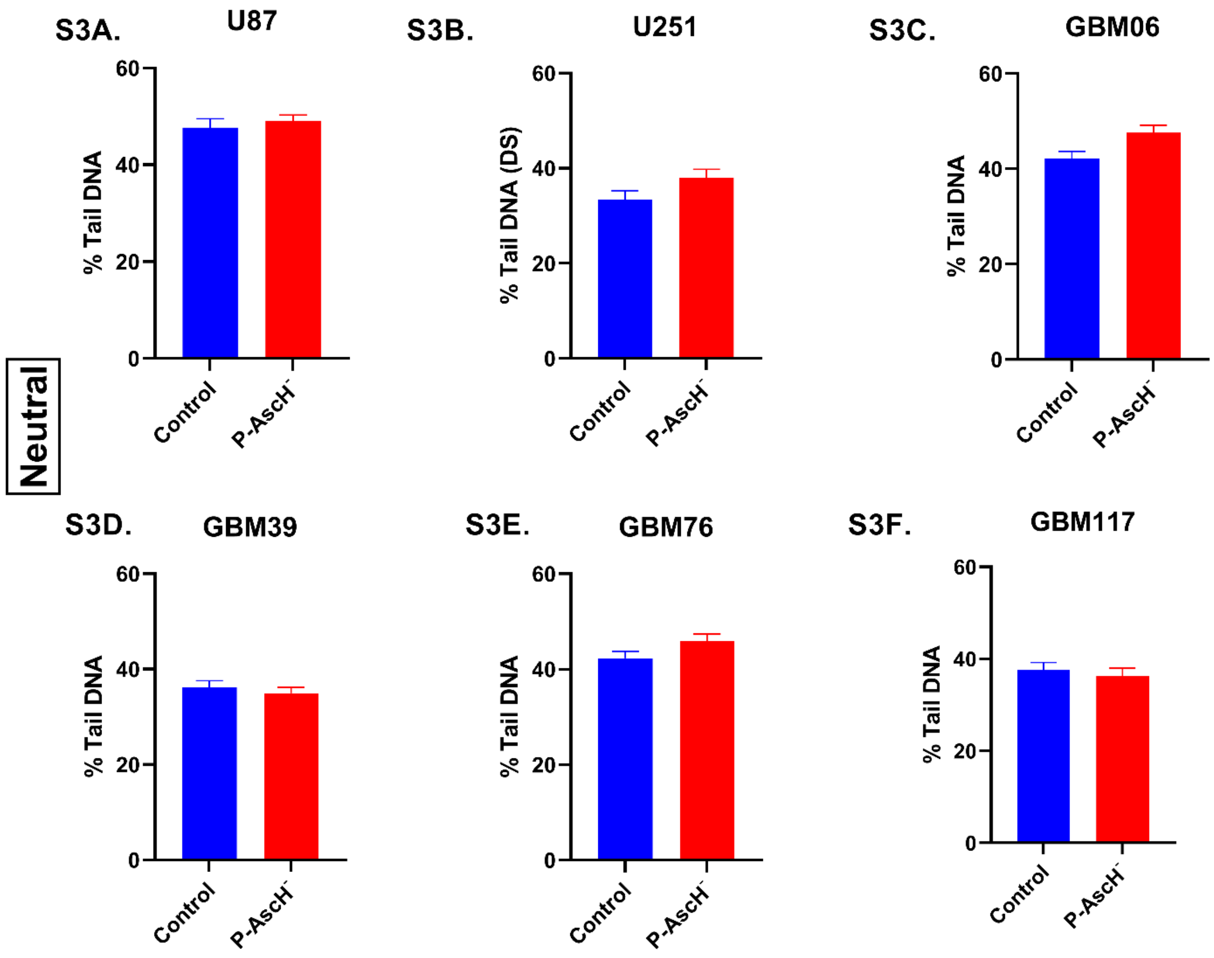

Figure S3. Double-strand DNA damage following P-AscH$^-$ treatment in GBM cells. Double strand DNA damage as % Tail DNA following a neural comet assay in GBM cells treated with 20 pmol/cell P-AscH- for 1 hour. Data are representing 3 replicates in each cell line (n = 3). Error bars represent standard error of the mean (SEM).

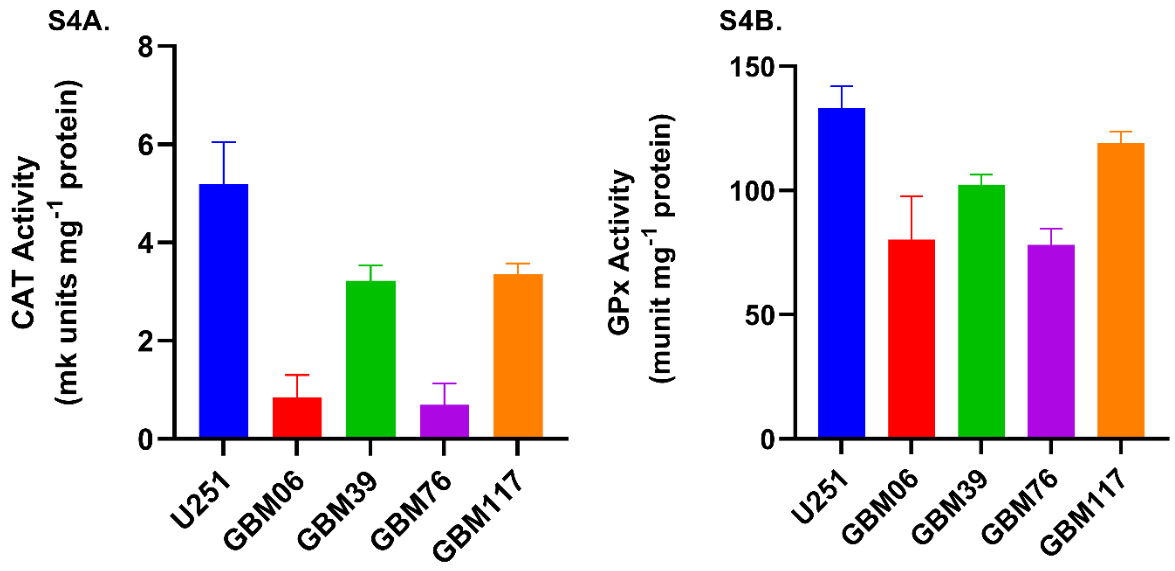

Figure S4. Catalase and glutathione peroxidase (GPx) enzyme activities in GBM cells. S4A. Catalase activity (mk units per mg protein) in GBM cells. **S4B.** GPx1 activity (munit per mg protein) in GBM cells. Data are representing 3 replicates in each cell line (n = 3). Error bars represent standard error of the mean (SEM).

Table S1. Hydrogen peroxide (H_2O_2) removal capacities among GBM cell lines of different subtypes. H_2O_2 removal rates (10^{-12} s^{-1} $cell^{-1}$ L) in GBM cells ± the standard deviation. Data are representing 3 replicates in each cell line (n = 3).

**Cell Line**	**Subtype**	**Avg. H_2O_2 Removal Rate kcell (10^{-12} s^{-1} $cell^{-1}$ L)**	**± SD**
GBM 06	Classical	3.68	± 1.5
GBM 76	Classical	5.2	± 1.9
GBM 39	Mesenchymal	1.76	± 0.9
U87	Mesenchymal	5.66	± 2.1
U251	Mesenchymal	11.7	± 4.0
GBM85	Proneural	23.6	± 5.1
GBM117	Proneural	18.0	± 2.5
